# Quantum Random Number Generation Using a Quanta Image Sensor

**DOI:** 10.3390/s16071002

**Published:** 2016-06-29

**Authors:** Emna Amri, Yacine Felk, Damien Stucki, Jiaju Ma, Eric R. Fossum

**Affiliations:** 1 ID Quantique SA, Ch. de la Marbrerie 3, 1227 Carouge, Switzerland; yacine.felk@idquantique.com (Y.F.); damien.stucki@idquantique.com (D.S.); 2 Thayer Engineering School at Dartmouth College, Hanover, NH, USA; Jiaju.Ma.TH@dartmouth.edu (J.M.); eric.r.fossum@dartmouth.edu (E.R.F.)

**Keywords:** QRNG, random number generator, QIS, quanta image sensor, photon counting, jot, entropy, randomness

## Abstract

A new quantum random number generation method is proposed. The method is based on the randomness of the photon emission process and the single photon counting capability of the Quanta Image Sensor (QIS). It has the potential to generate high-quality random numbers with remarkable data output rate. In this paper, the principle of photon statistics and theory of entropy are discussed. Sample data were collected with QIS jot device, and its randomness quality was analyzed. The randomness assessment method and results are discussed.

## 1. Introduction

The generation of high-quality random numbers is becoming more and more important for several applications such as cryptography, scientific calculations (Monte-Carlo numerical simulations) and gambling. With the expansion of computers’ fields of use and the rapid development of electronic communication networks, the number of such applications has been growing quickly. Cryptography, for example, is one of the most demanding applications. It consists of algorithms and protocols that can be used to ensure the confidentiality, the authenticity and the integrity of communications and it requires true random numbers to generate the keys to be used for encoding. However, high-quality random numbers cannot be obtained with deterministic algorithms (pseudo random number generator); instead, we can rely on an actual physical process to generate numbers. The most reliable processes are quantum physical processes which are fundamentally random. In fact, the intrinsic randomness of subatomic particles’ behavior at the quantum level is one of the few completely random processes in nature. By tying the outcome of a random number generator (RNG) to the random behavior of a quantum particle, it is possible to guarantee a truly unbiased and unpredictable system that we call a Quantum Random Number Generator (QRNG). 

Several hardware solutions have been used for true random number generation, and some of them are exploiting randomness in photon emission process. This class of QRNG includes beam splitters and single-photon avalanche diodes (SPADs) [1,2,3], homodyne detection mechanisms [4,5] and conventional CMOS image sensors (CIS) [6]. Although it has been demonstrated that these devices produce data of a satisfactory randomness quality, more work needs to be done to enhance the generation process, especially on the improvement of output data rate and device scalability. Practically, in an RNG utilizing image sensors, the photon emission is not the only source of randomness, and some noise sources in the detector, such as dark current and 1/*f* noise, will act as extra randomness sources and reduce the randomness quality since they have a strong thermal dependency. Therefore, an ideal detector should have high photon-counting accuracy with low read noise and low dark current to completely realize quantum-based randomness.

The Quanta Image Sensor (QIS) can be regarded as a possible solution to meet these goals because of its high-accuracy photon-counting capability, high output-data rate, small pixel-device size, and strong compatibility with the CIS fabrication process.

Proposed in 2005 as a “digital film sensor” [7], QIS can consist of over one billion pixels. Each pixel in QIS is called a “jot”. A jot may have sub-micron pitch, and is specialized for photon-counting capability. A QIS with hundreds of millions of jots will work at high speed, e.g., 1000 fps, with extremely low power consumption, e.g., 2.5 pJ/bit [8]. In each frame, each jot counts incident photons and outputs single-bit or multi-bit digital signal reflecting the number of photoelectrons [9]. The realization of QIS concept relies on the photon-counting capability of a jot device. As photons are quantized particles in nature, the signal generated by photons is also naturally quantized. However, with the presence of noise in the read out electronics, the quantization effect is weakened or eliminated. To realize photon-counting capability, deep sub-electron read noise (DSERN) is a prerequisite, which refers to read noise less than 0.5 e− r.m.s. But, high-accuracy photon-counting requires read noise of 0.15 e− r.m.s. or lower [10,11].

The pump-gate (PG) jot device designed by the Dartmouth group achieved 0.22 e− r.m.s. read noise with single correlated double sampling (CDS) read out at room temperature [12,13]. The low read noise of PG jot devices was fulfilled with improvements in conversion gain (CG) [14], and the photoelectron counting capability was demonstrated with quantization effects in the photon counting histogram (PCH) [15]. 

## 2. Randomness Generation Concept

To quantify the randomness in a sequence of bits, we refer to the concept of entropy, first introduced by Shannon [16]. Entropy measures the uncertainty associated with a random variable and is expressed in bits. For instance, a fair coin toss has an entropy of 1 bit, as the exact outcome—head or tail—cannot be predicted. If the coin is unfair, the uncertainty is lower and so is the entropy. And when tossing a two-headed coin, there is no uncertainty which leads to 0 bit of entropy.

To compute the value of the entropy, we need to have full information about the random number generation process. In a photon source, the photon emission process obeys the principle of Poisson statistics [10], and the probability P[k] of k photoelectron arrivals in a QIS jot is given by:

(1) $$P\left[k\right]=\frac{e^{-H}H^{k}}{k!}$$

where the quanta exposure H is defined as the average number of photoelectrons collected in each jot per frame. So under the illumination of a stable light source, randomness exists in the number of photoelectrons arriving in each frame.

During readout, the photoelectron signal from the jot is both converted to a voltage signal through the conversion gain (V/e−) and corrupted by noise. Let the readout signal *U* be normalized by the conversion gain and thus measured in electrons. The readout signal probability distribution function (PDF) becomes a convolution of the Poisson distribution for quanta exposure *H* and a normal distribution with read noise *u_n_* (e− r.m.s.). The result is a sum of constituent PDF components, one for each possible value of *k* and weighted by the Poisson probability for that *k* [11]:

(2) $$P\left[U\right]=\overset{\infty }{\underset{k=0}{\sum }}\frac{1}{\sqrt{2\pi u_{n}^{2}}}\text{exp}\left[-\frac{{\left(U-k\right)}^{2}}{2u_{n}^{2}}\right]\cdot \frac{e^{-H}H^{k}}{k!}$$

**Figure 1:**
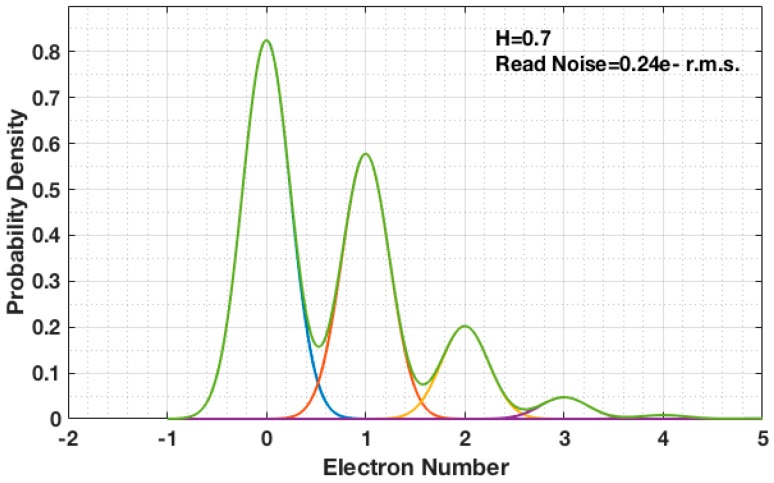
Readout signal probability distribution function (PDF) from Poisson distribution corrupted with read noise. Quanta exposure H = 0.7 and read noise u_n_ = 0.24 e− r.m.s.

An example of a Poisson distribution corrupted with read noise is shown in Figure 1. While in practice the photodetector may be sensitive to multiple photoelectrons, subsequent circuitry can be used to discriminate the output to two binary states (either a “0” meaning no photoelectron, or a “1” meaning at least one photoelectron) by setting a threshold $U_{t}$ between 0 and 1, typically 0.5 and comparing *U* to this threshold. From a stability perspective, it is better to choose the threshold $U_{t}$ at a valley of the readout signal PDF, such as at a 0.50 e− when H = 0.7, so that small fluctuations in light intensity have minimal impact on the value of entropy. The probability of the “0” state is given by:

(3) $$P\left[U<U_{t}\right]=\overset{\infty }{\underset{k=0}{\sum }}\frac{1}{2}\left[1+\text{erf}\left(\frac{U_{t}-k}{u_{n}\sqrt{2}}\right)\right]\cdot \frac{e^{-H}H^{k}}{k!}$$

and the probability of the “1” state is just:

(4) $$P\left[U\geq U_{t}\right]=1-P[U<U_{t}]$$

The minimum quantum entropy of this distribution is given by [6]:

(5) $$S_{min}=-\log_{2}[\text{max}(P\left[U\geq U_{t}\right],P[U<U_{t}])]$$

**Figure 2:**
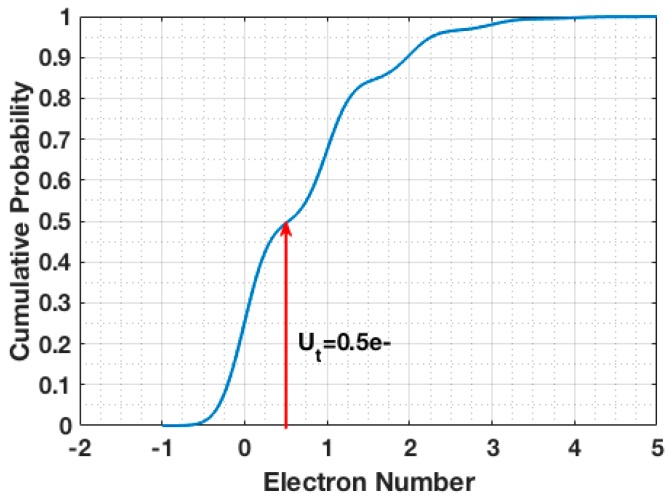
Cumulative probability of readout signal with read noise *u_n_* = 0.24 e− r.m.s. and quanta exposure *H* = 0.7.

If the measured value *U* will be encoded over *b* bits, the quantum entropy per bit of output will be, on average, equal to:

(6) $$\bar{S}=\frac{S_{min}}{b}<1$$

where *b* = 1 for the single-bit QIS. It is, therefore, optimal to choose a quanta exposure *H* such that $P\left[U<U_{t}\right]\;$=$\;P\left[U\geq U_{t}\right]\;$= 0.5. These two conditions of stability and entropy lead to a preferred quanta exposure H≅0.7. An example of the cumulative probability function for the readout signal for *H* = 0.7 is shown in Figure 2. It should be noted that other combinations of *H* and $U_{t}$ such as *H* = 2.67 and $U_{t}\;$= 2.5 e− are also viable options. For read noise *u_n_* above 1 e− r.m.s., where the photon-counting peaks of Figure 1 are fully “blurred” by noise (e.g., conventional CMOS image sensors), the optimum settings of $U_{t}$ and *H* converge so that the resultant Gaussian readout signal PDF is split in half at the peak, as one might deduce intuitively. 

**Figure 3:**
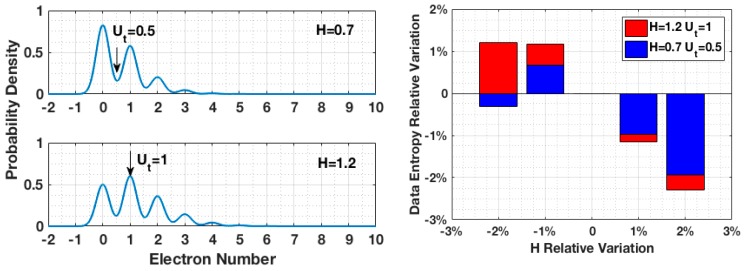
Binary data entropy variation caused by quanta exposure fluctuation during data collection.

Stability is illustrated by comparing two cases with different quanta exposures and respective thresholds: *H* = 0.7 and *H* = 1.2. As shown in Figure 3, the thresholds for each case were selected to maximize the binary data entropy: $U_{t}$ = 0.5 is located at a valley of PCH for *H* = 0.7, and $U_{t}$ = 1 is located at a peak of PCH for *H* = 1.2. With 2% variation of quanta exposure in both cases, the output data of *H* = 0.7 showed better stability in entropy.

It should be noted that only perfectly random bits will have unity quantum entropy, otherwise an extractor is required. A randomness extractor is a mathematical tool used to post-process an imperfect sequence of random bits (with an entropy less than 1) into a compressed but more random sequence. The quality of a randomness extractor is defined by the probability that the output deviates from a perfectly uniform bit string. This probability can be made arbitrarily very small by increasing the compression factor. The value of this factor depends on the entropy of the raw sequence and the targeted deviation probability and must be adjusted accordingly.

In this paper, we used a non-deterministic randomness extractor based on Universal-2 hash functions [17]. This extractor computes a number *q* of high-entropy output bits from a number *n > q* of lower-entropy (raw) input bits. This is done by performing a vector-matrix multiplication between the vector formed by the raw bit values and a random *n* x *q* matrix *M* generated using multiple entropy sources. The compression ratio is thus equal to the number of lines divided by the number of columns of *M*. After extraction, statistical tests are run in order to make sure that randomness specifications are fulfilled.

## 3. Data Collection

**Figure 4:**
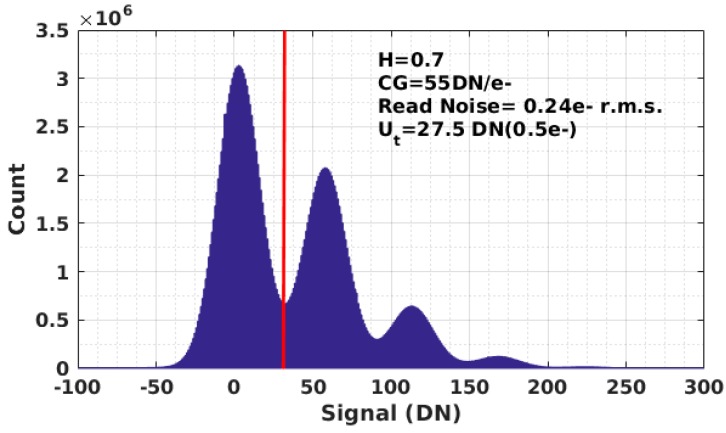
Photon counting histogram (PCH) of the first 200,000,000 samples.

**Figure 5:**
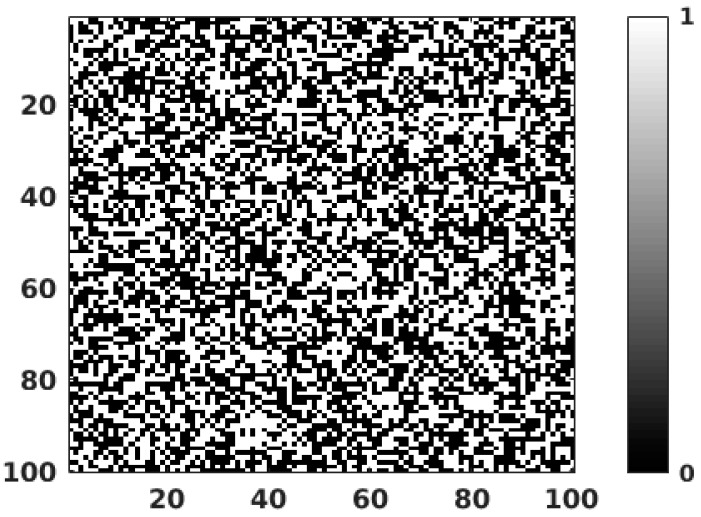
The binary output of the first 10,000 samples.

The feasibility of applying the QIS to the QRNG application was tested with PG jot devices. In the PG jot test chip, an analog readout approach is adopted. The output signal from 32 columns is selected by a multiplexer and then amplified by a switch-capacitor programmable gain amplifier (PGA) with a gain of 24. The output signal from the PGA is sent off-chip and digitized through a digital CDS implemented with an off-chip 14-bit ADC. A complete description of readout electronics can be found in [13]. A 3 × 3 array of green LEDs was used as light source, located in front of the test chip. The distance from the light source to the sensor was 2 cm, and the intensity of the light source was controlled by a precision voltage source. During the data collection, a single jot with 0.24 e− r.m.s. read noise was selected and read out repeatedly, and a 14-bit raw digital output was collected. Under the limitation of the readout electronics on this test chip, the single jot was readout at a speed of 10 ksample/s. The testing environment was calibrated with 20,000 testing samples, and the quanta exposure H was obtained using the PCH method. In order to improve the randomness entropy of the data, the threshold $U_{t}$ was determined as the median of the testing samples and then used with later samples to generate binary random numbers. The experimental PCH created by 200,000,000 samples is shown in Figure 4, which shows quanta exposure *H* of 0.7, and a read noise of 0.24 e− r.m.s. The threshold was set to 27.5DN, or 0.5 e−. The binary random numbers generated by first 10,000 samples are shown in Figure 5.

**Figure 6:**
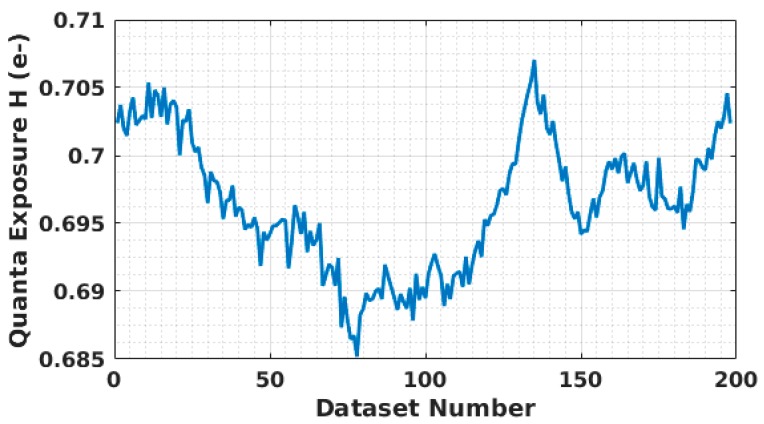
Quanta exposure fluctuation during data collection. Each dataset contains 1,000,000 samples.

Although the light source was controlled by a stable voltage source, there was still a small fluctuation inferred in the light intensity. As shown in Figure 6, the quanta exposure *H* of 200 datasets is depicted, in which each dataset contains 1,000,000 samples and *H* is determined for each data set using its PCH. During the data collection, about 2.1% variation in quanta exposure was observed. To minimize the impact of light source fluctuation, the testing environment was calibrated to have an average quanta exposure *H* close to 0.7, for which the threshold $U_{t}$ is located at a valley between two quantized peaks in the PCH.

## 4. Results

For a first test, we collected 500 Mbyte of raw random numbers by reading the jot at 5 ksamples/s (200 h of data collection). Using Equation (5), we were able to compute a minimum quantum entropy per output bit equal to 0.9845 for *H* = 0.7 and *u_n_* = 0.24 e− r.m.s. Then we used the obtained value in the formula of the probability that the extractor output will deviate from a perfectly uniform q-bit string:

(7) $$\varepsilon_{hash}=2^{-\left(\bar{S}n-m\right)/2}$$

where *n* is the number of raw bits and *m* the number of extracted random bits.

Since a value of $\varepsilon_{hash}=0$ is generally unachievable, we try to keep $\varepsilon_{hash}$ below $2^{-100\;}$ implying that even using millions of jots one will not see any deviation from perfect uniform randomness in a time longer than the age of the universe. This gave a compression factor for *n* = 1024 equal to 1.23 which corresponds to losing only 18% of the input raw bits.

After extraction, we perform NIST tests [18] on the obtained random bits. This set of statistical tests evaluate inter alia, the proportion of 0 s and 1 s in the entire sequence, the presence of periodic or non-periodic patterns and the possibility of compression without loss of information. The QIS-based QRNG passed all these tests.

## 5. Comparison with Other Technologies

The idea of using an optical detector for random number generation is not new and has been driven by the intrinsic quantum nature of light. Single Photon Avalanche Diode (SPAD) arrays illuminated by a photon source and operating in Geiger mode have been widely used for this purpose [19,20]. Besides the single photon detection capability and technology maturity, SPAD matrices offer high-quality random data and can be fabricated in standard CMOS manufacturing line. However, these SPAD sensors require high supply voltage (22–27 V) for biasing above breakdown, suffer from after-pulsing phenomena, and have lower throughput per unit area than other optical detectors because of larger pixel size (600 Mbits/s for a matrix size of $2.5\;{\mathrm{mm}}^{2}$ [19] and 200 Mbits/s for a matrix size of $3.2\;{\mathrm{mm}}^{2}$ [20]).

Another technology exploiting optical quantum process has been recently introduced by the University of Geneva [6] and it consists of extracting random numbers of a quantum origin from an illuminated CIS. This low-power technology is more compatible with consumer and portable electronics since cameras are currently integrated in many common devices. Unfortunately, conventional image sensors are not capable of single-photon detection and provide lower randomness quality [6], which requires higher compression factor and hence lower output data rate. The choice of using QIS for random number generation was driven by the results obtained with SPADs and CIS since we noticed that QIS covers the advantages of both technologies (best tradeoff between data rate and scalability, single photon detection and CMOS manufacturing line) while providing solutions for most of their problems (speed, dark count rate, detection efficiency). Table 1 summarizes the comparison of the three techniques performances under the assumption of being used as RNGs. Note that the generation processes are different which limits the comparison points. 

## 6. Summary

A new quantum random number generation method based on the QIS is proposed. Taking advantage of the randomness in photon emission and the photon counting capability of the Quanta Image Sensor, it shows promising advantages over previous QRNG technologies. Testing data was collected with QIS pump-gate jot device, and the randomness quality was assessed. Both randomness assessment method and data collection process are discussed, and the results show good randomness quality. 

## Figures and Tables

**Table 1:** The three technologies main comparison points.

Criteria	QIS	CIS	SPADs Matrix
Data Rate ^1^	5–12 Gb/s	0.3–1 Gb/s	0.1–0.6 Gb/s
Read Noise	<0.25 e− r.m.s.	>1 e− r.m.s.	<0.15 e− r.m.s.
Dark Current/Count Rate ^2^	0.1 e−/(jot·s)	10–500 e−/(pix·s)	200 counts/(pix·s)
Power Supply	2.5/3.3 V	2.5/3.3/5 V	22–27 V
Single Photon Counting	YES	NO	YES

^1^ For a device with 2.5 mm^2^ area size; ^2^ We define Dark Current for QIS/CIS and Dark Count Rate for SPADs, these values are measured at room temperature.
